# Bayesian additive tree ensembles for composite quantile regressions

**DOI:** 10.1007/s11222-025-10711-w

**Published:** 2025-08-26

**Authors:** Yaeji Lim, Ruijin Lu, Madeleine St. Ville, Zhen Chen

**Affiliations:** 1 Department of Applied Statistics, Chung-Ang University, Seoul, Korea; 2 Institute for Informatics, Data Science & Biostatistics, Washington University in St. Louis, Missouri, USA; 3 Eunice Kennedy Shriver National Institute of Child Health and Human Development, National Institutes of Health, Maryland, USA

**Keywords:** Bayesian additive regression trees, Composite quantile regression, Heavy-tailed errors, Non-linear covariate effects

## Abstract

In this paper, we introduce a novel approach that integrates Bayesian additive regression trees (BART) with the composite quantile regression (CQR) framework, creating a robust method for modeling complex relationships between predictors and outcomes under various error distributions. Unlike traditional quantile regression, which focuses on specific quantile levels, our proposed method, composite quantile BART, offers greater flexibility in capturing the entire conditional distribution of the response variable. By leveraging the strengths of BART and CQR, the proposed method provides enhanced predictive performance, especially in the presence of heavy-tailed errors and non-linear covariate effects. Numerical studies confirm that the proposed composite quantile BART method generally outperforms classical BART, quantile BART, and composite quantile linear regression models in terms of RMSE, especially under heavy-tailed or contaminated error distributions. Notably, under contaminated normal errors, it reduces RMSE by approximately 17% compared to composite quantile regression, and by 27% compared to classical BART.

## Introduction

Quantile methods are popular approaches for handling non-Gaussian data in regression modeling framework (Koenker and Hallock 2001; Hao and Naiman 2007). Unlike ordinary least squares (OLS) regression that focuses on the mean outcome given predictor variables, quantile regression (QR) examines the effects of covariates on the entire distribution of the response variable, offering a more comprehensive characterization of the data. This method provides robust results in the presence of heavy-tailed errors or outliers (Koenker 2005), making it a valuable tool for many applied settings. QR models have been successfully applied in epidemiology (Lee and Neocleous 2010; Wei et al. 2019), climatology (Haugen et al. 2018; Reich et al. 2011), and economics (Fitzenberger et al. 2013; Marrocu et al. 2015).

Except in situations where a particular quantile level is of interest (e.g., growth percentiles (Wei et al. 2006; Chen and Müller 2012)), the choice of appropriate quantile levels in quantile analysis affects the relative efficiency of the estimators, presenting a challenge in practical applications (Koenker and Bassett Jr 1978; Zhao and Xiao 2014). Zheng et al. (2015) also highlighted challenges in traditional quantile regression approaches that focus on estimating covariate effects at a single or a few prespecified quantile levels, noting the lack of a clear scientific basis for choosing one quantile over a nearby alternative. Additionally, it has been shown that QR can have arbitrarily low relative efficiency compared to OLS. To overcome the drawbacks of traditional QR, Zou and Yuan (2008) proposed a composite quantile regression (CQR) method to address multiple quantile regression models concurrently. Since then, significant efforts have been made to extend CQR. Jiang et al. (2012) pointed out that applying the same weight across different quantile levels is typically suboptimal and introduced weighted CQR (WCQR), which was later enhanced by Zhao and Lian (2016) to improve its efficiency. Unlike traditional QR, CQR does not require selecting specific quantiles and retains the robustness and other desirable properties of the quantile method (Zhao and Xiao 2014). In addition, when the error variance is finite, CQR still enjoys great advantages in terms of estimation efficiency. This approach enhances the flexibility and efficiency of the QR framework, allowing for a more holistic analysis of the response variable’s distribution. Huang and Chen (2015), Xu et al. (2017), and Yuan et al. (2023) are a few examples where CQR have been shown to outperform regular QR.

Both frequentist and Bayesian approaches to QR and CQR are abundant. Under the frequentist framework, Taylor (2000) proposed a quantile regression neural network (QRNN) to estimate the conditional probability distribution of multiperiod financial returns, and Jiang et al. (2013) extended a CQR estimation procedure for single index models. Galvao and Kato (2016) and Powell (2022) developed QR models for panel data. Bayesian approaches to QR make use of the equivalence between the minimization of the loss with the quantile check function of Koenker (2005) and maximization of likelihood function with an asymmetric Laplace distribution (ALD) error term (Yu and Moyeed 2001; Sriram et al. 2013). Using the mixture representation proposed by Kozumi and Kobayashi (2011), a Gibbs sampler for Bayesian QR can be implemented. This approach facilitates the estimation of complex models and the incorporation of prior information, enhancing the overall inferential process. For example, Li et al. (2010) explored regularization in Bayesian QR, while Yang et al. (2016) established the asymptotic validity of posterior inference for pseudo-Bayesian QR methods using an asymmetric Laplace likelihood. Additionally, Benoit and Van den Poel (2017) developed an R package for estimating QR parameters through a Bayesian approach based on the asymmetric Laplace distribution. These developments illustrate the potential of Bayesian methods to address various challenges in QR, including model complexity and computational efficiency. For the composite quantile model, Huang and Chen (2015) proposed a WCQR model where the weight of each component can be treated as an unknown parameter and estimated via Markov chain Monte Carlo (MCMC) sampling in a Bayesian hierarchical framework. This approach allows for greater flexibility in modeling and can lead to improved predictive performance by optimally combining information from multiple quantiles.

In many applications, the linearity assumption between covariates and conditional quantiles might not hold. In these situations, semi- or nonparametric approaches are attractive alternatives. For example, Koenker et al. (1994) considered regression spline approaches for estimating the conditional QR and Yu and Jones (1998) proposed local linear polynomial QR. Similarly, Kai et al. (2010) developed the local polynomial CQR estimators and proved its efficiency for non-normal error distributions. For Bayesian approaches, Thompson et al. (2010) proposed a nonparametric Bayesian QR method using natural cubic splines, offering a flexible alternative to parametric Bayesian QR models, particularly when the linearity assumption fails. More recently, Xu and Reich (2023) proposed a nonlinear simultaneous QR model by specifying a Bayesian nonparametric model for the conditional distribution.

Recently, Bayesian regression trees and their ensembles have demonstrated enhanced predictive performance in least squares regression, binary classification, and multiclass classification contexts. These methods have garnered significant attention due to their flexibility and ability to model complex relationships between variables. Notably, Bayesian additive regression trees (BART) (Chipman et al. 2012) estimate the conditional mean of a response given a set of predictors using a sum of regression trees model, showing remarkable predictive performance across various applications (Sparapani et al. 2016; Zhang et al. 2020). This approach leverages the power of multiple regression trees to capture non-linear interactions and intricate patterns in data, making it a robust tool for various statistical modeling tasks. Furthermore, Linero (2018) demonstrated the utility of the Dirichlet splitting probability prior within the BART framework for both prediction and variable selection problems. Additionally, Linero and Yang (2018) introduced soft decision trees and sparsity-inducing priors in BART, illustrating their promising performance. Several extensions of BART have been proposed to handle different types of outcomes. Notably, Kindo (2016) developed BART-based methods for multinomial, ordinal, and quantile regression as part of his dissertation. Subsequent works further advanced these directions, including multinomial probit BART (Xu et al. 2024) and BART for ordinal outcomes (Lee and Hwang 2024). Basak et al. (2022) developed BART for censored survival data, and Um et al. (2023) extended BART to multivariate skewed responses. These extensions have broadened the applicability of BART, enabling it to address a wider range of statistical challenges beyond traditional regression. Although BART has primarily been used for mean regression, several recent studies have extended it to quantile or tail estimation. For example, Clark et al. (2023) used BART-based vector autoregressions to perform real-time tail forecasting of GDP growth, inflation, and unemployment. In addition, Clark et al. (2024) employed a nonparametric quantile panel regression model that uses BART to capture nonlinear effects, and Baumeister et al. (2024) proposed a mixture BART model with stochastic volatility for forecasting the tails of the conditional distribution. In contrast to these applications, Kindo et al. (2016) developed a quantile version of BART (QBART) by incorporating the asymmetric Laplace distribution into the model, allowing for direct estimation of conditional quantiles. They demonstrated its superiority over linear quantile regression and quantile random forests.

In this paper, we consider the integration of BART into the composite quantile method, creating a BART for composite quantile regression (BART-CQR) to improve prediction accuracy under a wide range of error distributions. Rather than targeting a specific conditional quantile, BART-CQR aims to produce robust and efficient estimates of the conditional mean by aggregating information across multiple quantile levels. This aligns with the original motivation of CQR (Zou and Yuan 2008), which seeks robustness against heavy-tailed and non-Gaussian errors while maintaining estimation efficiency. Related work by Cao et al. (2024) proposed an adaptive trimmed regression approach based on BART, which enhances robustness by incorporating data-driven tuning parameters. However, their method focuses on effectively identifying suspected outliers and removing them from the analysis. In contrast, our BART-CQR method takes a different approach to robustness: rather than detecting and trimming outliers, it leverages the composite quantile framework, which inherently provides robustness to distributional misspecification without excluding any observations. This design enables BART-CQR to utilize the full dataset while maintaining efficiency and robustness in estimating the conditional mean.

Our approach extends the work of QBART by Kindo et al. (2016), which focused on single quantile regression, by generalizing it to composite quantile regression. Therefore, our focus is on accurate and robust prediction of the conditional mean, not on modeling specific quantiles. Our method also builds upon existing Bayesian composite quantile methods (Huang and Chen 2015; Alhamzawi 2016), which are based on linear models, by introducing a flexible additive regression tree framework to better capture nonlinear relationships between outcomes and predictors. This model is a fully Bayesian framework for constructing composite quantile regression trees and their ensembles. Through numerical studies, we verify that the BART-CQR outperforms the classical BART, QBART and CQR models under heavy-tailed distributions and in the presence of non-linearity between the input and output variables. This indicates that the proposed method combines the benefits of ensemble trees and the composite quantile approach, offering a powerful tool for handling complex and heavy-tailed data distributions.

The rest of the article is organized as follows. Section 2 introduces the BART-CQR method in detail, explaining its underlying principles and key features. Section 3 provides details on the posterior sampling of BART-CQR. Section 4 presents simulation results, and Section 5 illustrates a real data application. Conclusion and discussion are presented in Section 6. The R codes for implementing the numerical experiments in this study are available at https://github.com/yaeji-lim/BARTCQR.

## BART for composite quantile regressions

### Bayesian QR and CQR

Given $(y_i, \textbf{x} _i)$ for $i=1, \ldots , n,$ where $y_i \in {\mathbb {R}}$ and $\textbf{x} _i \in {\mathbb {R}}^p$, consider the following regression model:

1 $$\begin{aligned} y_i = \beta _0+ \textbf{x} _i^T {\varvec{\beta }}+ \epsilon _i, \end{aligned}$$

where $\beta _0$ is an intercept, ${\varvec{\beta }}= (\beta _1, \ldots , \beta _p)^T$ is the vector of unknown coefficients and $\epsilon _i \overset{\text {i.i.d.}}{\sim }ALD(\tau , \theta )$. The density function of $ALD(\tau , \theta )$ is $f_\theta (x| \tau ) = \theta (1- \theta ) \tau e^{- \tau \rho _\theta (x)}$, where $\theta \in (0,1)$ is an asymmetry parameter, $\tau$ is a precision parameter, and $\rho _\theta (t) = t (\theta - {\textbf {I}}_{(t < 0)})$ is the check loss function. Then, the conditional $\theta$th quantile of $y_i| \textbf{x} _i$ is

$$\begin{aligned} \beta _0+ \textbf{x} _i^T {\varvec{\beta }}+ q_{\epsilon _i} := b_\theta + \textbf{x} _i^T {\varvec{\beta }}, \end{aligned}$$

where $q_{\epsilon _i}$ is the $\theta$th quantile of $\epsilon _i$, and QR estimates coefficients by solving following minimization:

2 $$\begin{aligned} ({\hat{b}}_\theta , {\hat{{\varvec{\beta }}}})= \arg \min _{b_\theta , {\varvec{\beta }}} \sum _{i=1}^n \rho _{\theta } \left( y_i - b_\theta - \textbf{x} _i^T {\varvec{\beta }}\right) . \end{aligned}$$

The minimization is exactly equivalent to the maximization of a likelihood function,

3 $$\begin{aligned} \prod _{i=1}^n \Biggl \{ \theta (1- \theta ) \tau \exp \biggr [ - \tau \rho _{\theta }( y_i - b_\theta - \textbf{x} _i^T {\varvec{\beta }}) \biggr ] \Biggl \}. \end{aligned}$$

The minimum criterion (2) can be extended to the weighted CQR with multiple quantiles $\theta _1, \ldots , \theta _K$ as follows:

$$\begin{aligned} \arg \min _{ (b_{\theta _1},\ldots , b_{\theta _K}), {\varvec{\beta }}} \sum _{i=1}^n \sum _{k=1}^K \omega _k \rho _{\theta _k} \left( y_i - b_{\theta _k} - \textbf{x} _i^T {\varvec{\beta }}\right) , \end{aligned}$$

where $0 \le \omega _k \le 1$ is the weight for the *k*th component with $\sum _k \omega _k = 1$. We extend the joint distribution of (3) to the composite model as:

$$\begin{aligned} \prod _{i=1}^n \Biggr \{ \sum _{k=1}^K \omega _k \theta _k (1- \theta _k) \tau \exp \biggl [ - \tau \rho _{\theta _k}( y_i -b_{\theta _k} - \textbf{x} _i^T {\varvec{\beta }}) \biggl ] \Biggr \}. \end{aligned}$$

Due to the complexity of directly solving this, it is common to introduce a cluster matrix $\textbf{C}$, where the (*i*, *k*)th element, the latent variable $C_{ik}$, is equal to 1 if the *i*th subject belongs to the *k*th cluster; otherwise, $C_{ik}=0$. We assume that each observation belongs to exactly one cluster, i.e., for each *i*, $\sum _{k=1}^K C_{ik} = 1$. The complete likelihood is then:

4 $$\begin{aligned}&{ \prod _{i=1}^n [ P(y_i | \textbf{C} _i) \times P( \textbf{C} _i)] } \nonumber \\&\quad = \prod _{i=1}^n \prod _{k=1}^K \Biggr \{ \omega _k\theta _k (1-\theta _k) \exp \biggl [ - { \tau }\rho _{\theta _k}( y_i -b_{\theta _k} - \textbf{x} _i^T {\varvec{\beta }})\biggl ] \Biggr \} ^{C_{ik}}, \end{aligned}$$

where $$ \textbf{C} _i = (C_{i1}, \ldots , C_{iK})$$.

We place a Laplace prior on ${\varvec{\beta }}$ for regularization:

$$\begin{aligned} \pi ({\varvec{\beta }}|\tau ,\lambda ) = \left( \frac{ \tau \lambda }{2} \right) ^p \exp \left( - \tau \lambda \sum _{j=1}^p | \beta _j | \right) , \end{aligned}$$

and the prior can be further represented as

$$\begin{aligned} \pi ({\varvec{\beta }}|\eta ^2)= &  \prod _{j=1}^p \int _0^\infty \frac{1}{\sqrt{2 \pi s_j}} \exp \Biggr ( - \frac{ \beta _j^2}{2 s_j} \Biggr ) \frac{ \eta ^2}{2}\\ &  \times \exp \Biggr ( - \frac{ \eta ^2}{2} s_j \Biggr ) ds_j , \end{aligned}$$

where $\eta :=\tau \lambda$. For $\pi ({\varvec{\omega }})$, we assume $\pi ({\varvec{\omega }}) = \text { Dirichlet} (\alpha _1, \cdots , \alpha _K)$ with ${\varvec{\omega }}=(\omega _1, \ldots , \omega _K)^T$. The priors for $\tau$ and $\eta ^2$ are assumed to follow gamma distributions.

Then the posterior distribution is given by:

$$\begin{aligned}&\prod _{i=1}^n \prod _{k=1}^K \Biggr \{ \omega _k\theta _k (1-\theta _k) \exp \biggl [ -{ \tau } \rho _{\theta _k}( y_i -b_{\theta _k}- \textbf{x} _i^T {\varvec{\beta }})\biggl ] \Biggr \} ^{C_{ik}} \\&\quad \times \pi ( {\varvec{\beta }}| \eta ^2) { \pi (\tau , \eta ^2)} \pi ({\varvec{\omega }}). \end{aligned}$$

To obtain closed-form conditional distributions, we use the representation of ALD as a mixture of an exponential and a scaled normal distribution (Kozumi and Kobayashi 2011). The regression model (1) can then be expressed as:

$$\begin{aligned} y_i&= \beta _0 + \textbf{x} _i^T {\varvec{\beta }}+ \tau ^{-1} \xi _{1} \nu _i + \tau ^{-1}\xi _{2} \sqrt{ \nu _i} z_i \\&=\beta _0 + \textbf{x} _i^T {\varvec{\beta }}+ \xi _{1} {\tilde{\nu }}_i + \tau ^{-1/2}\xi _{2} \sqrt{ {\tilde{\nu }}_i} z_i , \end{aligned}$$

where $\xi _1 = \frac{1- 2 \theta }{\theta (1-\theta )}$ and $\xi _2^2 = \frac{2}{ \theta (1- \theta )}$. Here, $\nu _i \sim \exp (1)$, ${\tilde{\nu }}_i := \tau ^{-1} \nu _i \sim \exp (\tau ^{-1})$ and $z_i \sim N(0,1)$ are independent. The hierarchical model for MCMC sampling is then:

$$\begin{aligned} y_i =&\,b_{\theta _k} + \textbf{x} _i^T {\varvec{\beta }}+ \xi _{1k} {\tilde{\nu }}_i \\&\quad + \tau ^{-1/2}\xi _{2k} \sqrt{ {\tilde{\nu }}_i} z_i , ~~{ \text {for all } i~ \text {such that } C_{ik}=1}, \\ {\tilde{{\varvec{\nu }}}} | \tau \sim&\prod _{i=1}^n \tau \exp ( - \tau {\tilde{\nu }}_i) ~~~ \text {for} ~~{\tilde{{\varvec{\nu }}}}= ({\tilde{\nu }}_1, \ldots , {\tilde{\nu }}_n)^T,\\ \textbf{z} \sim&\prod _{i=1}^n \frac{1}{ \sqrt{2\pi }} \exp \Big ( -\frac{1}{2} z_i^2 \Big ) ~~~ \text {for} ~~ \textbf{z} = (z_1, \ldots , z_n)^T,\\ {\varvec{\beta }}, \textbf{s} | \eta ^2 \sim&\prod _{j=1}^p \frac{1}{\sqrt{2 \pi s_j}} \exp \Biggr ( - \frac{ \beta _j^2}{2 s_j} \Biggr ) \prod _{j=1}^p \frac{ \eta ^2}{2} \exp \Biggr ( - \frac{ \eta ^2}{2} s_j \Biggr ) \\&~~~ \text {for} ~~ \textbf{s} = (s_1, \ldots , s_p)^T,\\ ( \tau , \eta ^2) \sim&~ \tau ^{a_\tau -1} \exp (- b_\tau \tau ) ( \eta ^2)^{a_\eta -1} \exp (-b_\eta \eta ^2) ,\\ {\varvec{\omega }}\sim&\text { Dirichlet} (\alpha _1, \cdots , \alpha _K), \end{aligned}$$

where $a_\tau , b_\tau , a_\eta , b_\eta$ are hyperparameters.

Denote $\textbf{y} = (y_1, \ldots , y_n)^T$, $\textbf{X} = ( \textbf{x} _1, \ldots , \textbf{x} _n)^T$, and $\textbf{b} = (b_{\theta _1}, \ldots , b_{\theta _K} )^T$. The complete likelihood based on ALD form is:

$$\begin{aligned} f( \textbf{y} |&\textbf{X} , {\varvec{\beta }}, \textbf{s} , \eta ^2, \tau , {\tilde{{\varvec{\nu }}}}, \textbf{b} , {\varvec{\omega }}, \textbf{C} ) = \\&\prod _{i=1}^n \prod _{k=1}^K \Biggr ( \frac{1}{\sqrt{2\pi \tau ^{-1} \xi _{2k}^2 {\tilde{\nu }}_i} }\Biggr )^{C_{ik}}\\&\quad \exp \Biggr [ - \frac{1}{2} \sum _{i=1}^n \sum _{k=1}^K \frac{ C_{ik} ( y_i - b_{\theta _k} - \textbf{x} _i^T {\varvec{\beta }}- \xi _{1k} {\tilde{\nu }}_i )^2 }{ \tau ^{-1} \xi _{2k}^2 {\tilde{\nu }}_i} \Biggr ]. \end{aligned}$$

### BART CQR

To allow a semiparametric relationship between outcome and predictors, consider the following regression model:

5 $$\begin{aligned} y_i = h( \textbf{x} _i)+ \epsilon _i,~~~~~i=1, \ldots , n, \end{aligned}$$

where *h* is an unknown function, and $\epsilon _i \overset{\text {i.i.d.}}{\sim }ALD(\tau , \theta )$. The idea of applying BART to CQR is to model (5) as

$$\begin{aligned} y_i = b_{\theta _{k}}+ \sum _j g( x_i; T_j , M_j) + \epsilon _i,~~~{ \text {for all } i~ \text {such that } C_{ik}=1}, \end{aligned}$$

where $( \textbf{T} , \textbf{M} )= { (T_j, M_j); j=1, \ldots , n_T }$, with $T_j$ and $M_j$ being the parameters of the *j*th tree in the BART model.

We assume that the priors on any two distinct trees in the sum are independent and the prior on $\tau$ is independent of the tree priors. Further assuming that given a tree, the priors on its terminal node parameters are independent. Therefore,

$$\begin{aligned} p ( \textbf{T} , \textbf{M} , {\tilde{\nu }} , \tau )&= \bigg [\prod _{j=1}^{n_T} p(T_j, M_j) \bigg ] p({\tilde{\nu }} | \tau ) p(\tau )\\&= \bigg [\prod _{j=1}^{n_T} p(T_j) p( M_j| T_j) \bigg ] p({\tilde{\nu }} | \tau ) p(\tau )\\&= \Bigg [\prod _{j=1}^{n_T} \bigg [ p(T_j) \prod _{k=1}^{m_j} p( \mu _{jk}| T_j) \bigg ] \Bigg ] p({\tilde{\nu }} | \tau ) p(\tau ), \end{aligned}$$

where $M_j= (\mu _{j1}, \ldots , \mu _{jm_j})$, $m_j$ is the number of terminal nodes of tree $T_j$, and $n_T$ is the number of trees in the sum.

For the prior $p(T_j)$, we follow a *tree generating stochastic process* of Chipman et al. (2012).

$$\begin{aligned} P(\text {split at depth} ~ d) = {\left\{ \begin{array}{ll} 1 & d = 0 \\ \frac{\alpha _T}{(1+d)^{\beta _T}}, & d > 0, \end{array}\right. } \end{aligned}$$

where $\alpha _T \in (0,1)$, $\beta _T \in [0, \infty )$. In addition, we use uniform distribution for both of the distribution over the splitting variable and the distribution over the splitting rule.

Given a tree $T_j$, the prior on the terminal node parameters is a Gaussian distribution, $p( \mu _{jk} | T_j) \sim N( \mu _\mu , \sigma _\mu ^2 ),$ for $k=1, \ldots , m_j$. Hyper-parameters $\mu _\mu$ and $\sigma _\mu ^2$ are selected so that the overall effect induced by the prior distributions is in the interval $(y_{\min }, y_{\max })$ with high probability. As in Kindo et al. (2016), we use the transformation ${\tilde{y}} = \frac{ y - y_{\min } }{ y_{\max } - y_{\min } } - 0.5$, ensuring that the transformed response lies in the $(-0.5, 0.5)$ interval. Consequently, $p( \mu _{jk} | T_j) \sim N( 0 , \frac{1}{4\kappa ^2 {n_T} } ),$ for $k=1, \ldots , m_j$. We set $\kappa =2$, which has been found to yield good results, though it can be optimized through cross-validation.

Following standard Bayesian approach to CQR, we use the expression of ALD as a mixture of an exponential and a scaled normal distribution and obtain the following hierarchical models for BART-CQR:

6 $$\begin{aligned} y_i =&\, { b_{\theta _{k}}+ \sum _j g( x_i; T_j , M_j) } + \xi _{1k} {\tilde{\nu }}_i \nonumber \\&\quad + \tau ^{-1/2}\xi _{2k} \sqrt{ {\tilde{\nu }}_i} z_i, ~~{ \text {for all } i~ \text {such that } C_{ik}=1}, \nonumber \\ {\tilde{{\varvec{\nu }}}} | \tau \sim&\prod _{i=1}^n \tau \exp ( - \tau {\tilde{\nu }}_i), \nonumber \\ \tau \sim&~\tau ^{a_\tau -1} \exp (- b_\tau \tau ) , \nonumber \\ \textbf{z} \sim&\prod _{i=1}^n \frac{1}{ \sqrt{2\pi }} \exp \Big ( -\frac{1}{2} z_i^2 \Big ) ,\nonumber \\ {\varvec{\omega }}\sim&\text { Dirichlet} (\alpha _1, \cdots , \alpha _K) , \end{aligned}$$

where $$\xi _{1k} = \frac{1- 2\theta _k}{ \theta _k (1- \theta _k)}, $$ and $$\xi _{2k}^2 = \frac{2}{ \theta _k (1-\theta _k)}$$.

## Posterior Inference

The posterior computation is carried out using a Gibbs sampling algorithm. Based on the hierarchical model in (6), the posterior updating scheme proceeds through the following six steps:

$$\begin{aligned}&f( {\tilde{\nu }}_i | \textbf{T} , \textbf{M} , \textbf{Y} , \textbf{X} , \tau , {\tilde{\nu }}_{(-i)}, \textbf{b} , \textbf{C} ,{\varvec{\omega }}) ~~~\text {for}~~ i=1, \ldots , n\\&f( (T_j, M_j ) | \textbf{T} _{(-j)}, \textbf{M} _{(-j)}, \textbf{Y} , \textbf{X} , \tau , {\tilde{{\varvec{\nu }}}}, \textbf{b} , \textbf{C} ,{\varvec{\omega }})\\&~~~\text {for}~~ j=1, \ldots , n_T \\&f ( \tau | \textbf{T} , \textbf{M} , \textbf{Y} , \textbf{X} , {\tilde{{\varvec{\nu }}}}, \textbf{b} , \textbf{C} ,{\varvec{\omega }}) \\&f( \textbf{C} _i | \textbf{T} , \textbf{M} , \textbf{Y} , \textbf{X} , \tau , {\tilde{{\varvec{\nu }}}}, \textbf{b} , \textbf{C} _{-i} ,{\varvec{\omega }}) ~~~\text {for}~~ i=1, \ldots , n \\&f( {\varvec{\omega }}| \textbf{T} , \textbf{M} , \textbf{Y} , \textbf{X} , \tau , {\tilde{{\varvec{\nu }}}}, \textbf{b} , \textbf{C} ) \\&f( b_{\theta _k } | \textbf{T} , \textbf{M} , \textbf{Y} , \textbf{X} , \tau ,{\tilde{{\varvec{\nu }}}}, b_{-\theta _k } , \textbf{C} , {\varvec{\omega }}) ~~~\text {for}~~ k=1, \ldots , K. \end{aligned}$$

The full conditional distribution of ${\tilde{\nu }}_i$ follows a Generalized Inverse Gaussian distribution. The distribution of the precision parameter $\tau$ is Gamma, and the latent class assignments $\textbf{C} _i$ are drawn from a Multinomial distribution. For the mixture weights ${\varvec{\omega }}$, we use a Dirichlet distribution, and the intercept $b_{\theta _k}$ has a normal full conditional distribution.

The most challenging part is drawing the regression trees, $(T_j, M_j)$. Each pair is updated using a Bayesian backfitting strategy based on residuals from the other trees, similar to standard BART procedures. First define

7 $$\begin{aligned} &  r_{ij} := y_i -b_{\theta _{k}} - \sum _{l \ne j } g( \textbf{x} _i ; T_l , M_l ) - \xi _{1k} {\tilde{\nu }}_i = g( \textbf{x} _i ; T_j , M_j ) \nonumber \\ &  \quad \qquad \quad + \tau ^{-1/2}\xi _{2k} \sqrt{ {\tilde{\nu }}_i} z_i , \nonumber \\ &  \quad { \text {for all } i~ \text {such that } C_{ik}=1}. \end{aligned}$$

Then, draw from $(T_j, M_j)$ is equivalent to draw from a single regression tree $r_{ij}= g( \textbf{x} _i ; T_j , M_j ) + \tau ^{-1/2}\xi _{2k} \sqrt{ {\tilde{\nu }}_i} z_i$ for $i=1, \ldots , n$. In the tree $T_j$, assume that there are $m_j$ terminal nodes and that $n_{l}$ observations fall into terminal node *l* for $l=1, \ldots , m_j$ ($n_1 + \ldots + n_{m_j}=n$). Let’s consider the set of observations fall into terminal node *l*. Since for each $y_i$, there is corresponding $C_{ik}$ such that $C_{ik}=1$, we can consider a pair $\{(i, k) |~k \text { such that } C_{ik}=1 \}$ for each *i*. Denote the set of (*i*, *k*)’s that fall into terminal node *l* as $G_l$. Further, define $\textbf{r} _j = ( \textbf{r} _{j,1}, \ldots , \textbf{r} _{j,m_j})$ with $\textbf{r} _{j,l}= (r_{1, j, l } , \ldots , r_{n_l ,j,l})$, where $r_{i,j,l}$ be a observation that falls into *l*th terminal node in tree $T_j$ for $i \in G_l$, and we have $r_{i, j,l} \sim N ( \mu _{jl} , \tau ^{-1} \xi _{2k}^2 {\tilde{\nu }}_{i} )$ for $(i,k) \in G_l$. Then the likelihood of the single tree in (7) is

$$\begin{aligned} &  f( \textbf{r} _j | \textbf{X} , \textbf{T} _{j}, \textbf{M} _{j },\tau ,{\tilde{{\varvec{\nu }}}}, \textbf{b} , \textbf{C} , {\varvec{\omega }}) \\ &  \quad = \prod _{l=1}^{m_j} f( \textbf{r} _{j,l} | \textbf{X} _l , \textbf{T} _{j}, \textbf{M} _{j }, \tau ,{\tilde{{\varvec{\nu }}}}_l, \textbf{b} , \textbf{C} , {\varvec{\omega }}), \end{aligned}$$

where

$$\begin{aligned} &  f( \textbf{r} _{j,l} | \textbf{X} _l , \textbf{T} _{j}, \textbf{M} _{j }, \tau ,{\tilde{{\varvec{\nu }}}}_l, \textbf{b} , \textbf{C} , {\varvec{\omega }}) \\ &  \quad = \prod _{(i,k) \in G_l} \frac{1}{ \sqrt{2 \pi \tau ^{-1} \xi _{2k}^2 {\tilde{\nu }}_{i} }} \exp \bigg [ - \frac{1}{2} \frac{ (r_{i, j,l}- \mu _{jl} )^2 }{ \tau ^{-1} \xi _{2k}^2 {\tilde{\nu }}_{i}} \bigg ]. \end{aligned}$$

Now, the draw from $f( (T_j, M_j ) | \textbf{T} _{(-j)}, \textbf{M} _{(-j)}, \textbf{Y} , \textbf{X} , \tau , {\tilde{{\varvec{\nu }}}}, \textbf{b} , \textbf{C} , {\varvec{\omega }})$ can be done by two successive steps as

8 $$\begin{aligned} f( T_j&|&\textbf{r} _{j}, \tau , {\tilde{{\varvec{\nu }}}}, \textbf{b} , \textbf{C} , {\varvec{\omega }}) , \end{aligned}$$

9 $$\begin{aligned} f( M_j&|&T_j, \textbf{r} _{j}, \tau , {\tilde{{\varvec{\nu }}}} , \textbf{b} , \textbf{C} , {\varvec{\omega }}). \end{aligned}$$

For (8), we use Metropolis-Hastings algorithm. The formula of $f( T_j | \textbf{r} _{j}, \tau , {\tilde{{\varvec{\nu }}}}, \textbf{b} , \textbf{C} , {\varvec{\omega }})$ can be derived as

10 $$\begin{aligned}&f(T_j | \textbf{r} _{j}, \tau , {\tilde{{\varvec{\nu }}}}, \textbf{b} , \textbf{C} , {\varvec{\omega }}) \propto ~ p (T_j)\nonumber \\&\quad \int p( \textbf{r} _{j} | M_j , T_j, \tau , {\tilde{{\varvec{\nu }}}}, \textbf{b} , \textbf{C} , {\varvec{\omega }}) p(M_j | T_j , \tau , {\tilde{{\varvec{\nu }}}}, \textbf{b} , \textbf{C} , {\varvec{\omega }}) dM_j \nonumber \\&\quad = p (T_j) \prod _l \Biggl \{ \biggl ( \frac{1}{ \sqrt{ 2 \pi \tau ^{-1} }} \biggl )^{n_{l}} \biggl ( \prod _{(i,k) \in G_l} \xi _{2k}^{-1}{\tilde{\nu }}_{i}^{-1/2} \biggl ) \nonumber \\&\quad \exp \biggl ( - \frac{1}{2} \sum _{(i,k) \in G_l} \frac{ r_{i, j,l}^2 }{ \tau ^{-1} \xi _{2k}^2 {\tilde{\nu }}_{i}} \biggl )\nonumber \\&\quad \times \sqrt{ \frac{ \tau ^{-1} }{ \sigma _\mu ^2 \sum _{(i,k) \in G_l} {\tilde{\nu }}_{i}^{-1} \xi _{2k}^{-2} + \tau ^{-1} }}\nonumber \\&\quad \exp \biggl [ \frac{\sigma _\mu ^2 \biggl ( \sum _{(i,k) \in G_l} r_{i, j,l} {\tilde{\nu }}_{i}^{-1} \xi _{2k}^{-2} \biggl )^2 }{ 2\tau ^{-1} \biggl ( \tau ^{-1} + \sigma _\mu ^2 \sum _{(i,k) \in G_l} {\tilde{\nu }}_{i}^{-1} \xi _{2k}^{-2}\biggl ) } \biggl ] \Bigg \} . \end{aligned}$$

Given an update tree, $\mu _{jl}$, *l*th terminal node parameter at tree $T_j$ in (9) can be drawn as:

$$\begin{aligned}&f( \mu _{jl} | T_j, \textbf{r} _{j}, \tau , {\tilde{{\varvec{\nu }}}} , \textbf{b} , \textbf{C} , {\varvec{\omega }}) \propto \\&\quad \exp \Biggr [ - \biggl (\sum _{(i,k) \in G_l} \frac{ 1 }{{2 \tau ^{-1} \xi _{2k}^2 {\tilde{\nu }}_{i} } } + \frac{1}{ 2 \sigma _\mu ^2} \biggl ) \mu _{jl}^2 \nonumber \\&\quad + 2\sum _{(i,k) \in G_l} \frac{ r_{i,j,l} }{2 \tau ^{-1} \xi _{2k}^2 {\tilde{\nu }}_{i} } \mu _{jl} \Biggr ], \end{aligned}$$

which is a Gaussian distribution.

Complete derivations and the step-by-step sampling scheme are provided in Appendix A.

After running the algorithm sufficiently long after the burn-in period, we obtain a sequence of posterior draws of $f^*$;

$$\begin{aligned} f^*(x) = \sum _j g(x; T_j ^* , M_j^*), \end{aligned}$$

denoted as $f_1^*, \ldots , f_K^*$. The final estimate of *f*(*x*) at a given *x* is then taken as the average of $f_1^*, \ldots , f_K^*$, as in Chipman et al. (2012).

## Simulations

We consider Friedman’s five dimensional test function (Friedman 1991) to illustrate various features of the proposed method on simulated data. We construct data by simulating values of $\textbf{x} = (x_1, \ldots , x_p)$, where $x_1, \ldots , x_p~\overset{\text{ i.i.d. }}{\sim }~ \text{ Uniform } (0,1),$ and

$$\begin{aligned} y= &  f( \textbf{x} ) + \epsilon = 10 \sin (\pi x_1 x_2) + 20 (x_3 - 0.5)^2 \\ &  + 10 x_4 + 5x_5 + \epsilon . \end{aligned}$$

We consider various error distributions to demonstrate the superiority of the proposed method:

- Normal distribution: $$\epsilon \sim N(0,1)$$.
- Contaminated Normal distribution: $\epsilon = \epsilon _1 + \zeta _1 \epsilon _2 + \zeta _2 \epsilon _3$, where $\zeta _j \sim$ Bern(0.15),  $j=1,2$, being independent of $Y, \epsilon _1 , \epsilon _2$, and $\epsilon _3$, $\epsilon _1 \sim N(0,1)$, $\epsilon _2 \sim N(0,9^2),$ and $\epsilon _3 \sim ALD( \tau =1, \theta =0.9)$.
- *t*-distribution: $$\epsilon \sim t(2)$$.
- Asymmetric Laplace distribution: $\epsilon \sim ALD ( \tau =1 , \theta )$ with $\theta =0.1,~ 0.9$.

The model evaluation metric used is the root mean squared error (RMSE), given by

$$\begin{aligned} RMSE = \sqrt{\frac{1}{n} \sum _{i=1}^n (f( \textbf{x} _i)- {\hat{y}}_i)^2}, \end{aligned}$$

where $\textbf{x} _i= (x_{i1}, \ldots , x_{ip})$ is the *i*th covariate, $f( \textbf{x} _i)$ is the true regression function used to generate the data and ${\hat{y}}_i$ is the model’s prediction.

We compare the proposed composite quantile BART model (BART-CQR) with quantile BART (QBART) at quantile levels 25%, 50%, and 75% (Kindo et al. 2016), BART (Chipman et al. 2012), and composite quantile regression (CQ regression) (Huang and Chen 2015). For tree methods, we set the number of trees $n_T=200$, and for the composite quantile methods, we use $K=9$ quantile levels, $\theta _k= \frac{k}{K+1}$ for $k=1, \ldots , K$. For all methods, we set 3000 burn-in steps and use 8000 iterations.

We set $n=200$ and $p=30$ in each simulation and use 100 observations as the training set, with the remaining observations designated for the test set. We run 200 independent replications, and the test dataset performance is presented in Table 1.

**Table 1:** RMSE (standard error in parentheses) over 200 replications for Friedman’s five dimensional test function data with different error distributions. Bold face indicates the best performance

	BART-CQR	QBART (25%)	QBART (50%)	QBART (75%)	BART	CQ regression
Normal	1.800 (0.187)	2.641 (0.347)	2.736 (0.324)	2.644 (0.289)	**1.739 (0.175)**	2.591 (0.240)
Contaminated Normal	**2.712(0.427)**	8.285(1.630)	8.411(1.660)	7.276(1.487)	3.729(0.629)	3.245(0.440)
*t*(2)	**1.970(0.206)**	3.826(1.541)	4.076(1.320)	3.624(0.826)	2.325(0.811)	2.724(0.251)
ALD ($\theta =0.1)$	**9.317(1.347)**	14.228(2.105)	16.33(2.427)	16.774(2.501)	10.098(1.223)	9.689(1.181)
ALD ($\theta =0.9)$	**8.886(1.299)**	16.343(2.534)	15.976(2.67)	13.922(2.196)	9.862(1.236)	9.372(1.014)

The simulation results demonstrate that the BART-CQR model consistently outperforms other methods, particularly under heavy-tailed or skewed error distributions. As expected, classical BART performs best under normally distributed errors, where it is well suited; however, its performance deteriorates significantly under non-Gaussian settings. Notably, BART-CQR performs comparably well under normal errors, indicating its effectiveness even in well-behaved scenarios. Under the contaminated normal distribution and *t*(2) distribution, BART-CQR also yields the best test RMSE, highlighting its robustness. Finally, with the ALD at $\theta = 0.1$ and $\theta = 0.9$, BART-CQR maintains the lowest test RMSE, confirming its ability to handle complex and asymmetric error structures effectively. Interestingly, CQ regression outperforms QBART even though the underlying regression function is nonlinear. This may be attributed to the composite nature of the composite quantile loss, which aggregates information across multiple quantiles, thereby stabilizing estimation and reducing variance. In contrast, QBART focuses on individual quantiles, which may be more sensitive to noise and outliers, particularly under heavy-tailed or asymmetric error distributions.

To examine the sensitivity of BART-CQR to the prior specification on the error precision parameter $\tau$, we conducted a prior sensitivity analysis using three Gamma prior settings. The results, presented in Appendix B, indicate that the performance of BART-CQR remains robust across a wide range of prior choices.

We conduct additional simulations under the contaminated normal setting with increased training sample sizes to further evaluate the scalability of BART-CQR. The results, reported in Appendix C, show that BART-CQR’s performance improves as the sample size increases, consistently achieving the lowest RMSE compared to competitors.

We further investigate the effect of heteroscedastic errors and correlated input variables under the following model with $n=200$ and $p=30$:

$$\begin{aligned} y= 10 \sin (\pi x_1 x_2) + 20 (x_3 - 0.5)^2 + 10 x_4 + 5x_5 + \sigma ( \textbf{x} ) \epsilon , \end{aligned}$$

where $\textbf{x} := (x_1, \ldots , x_p)^T \sim N_p({\textbf {0}}, {\varvec{\Sigma }})$, ${\varvec{\Sigma }}= (\sigma _{ij})_{p \times p}$ with $\sigma _{ij}= 0.5^{|i-j|}$, and $\sigma ( \textbf{x} ) =\{ (1+ 2 \textbf{x} ^T) /3 \} {\textbf {1}}$, and $\epsilon \sim N(0,1)$. For each of the 200 replications, we randomly split the data into a training set of size 100 and a test set of size 100. The predictive performance was evaluated using the test set only. The results in Table 2 show that BART-CQR consistently achieves the lowest RMSE, demonstrating superior predictive performance under both heteroscedasticity and correlated covariates.

**Table 2:** RMSE (standard error in parentheses) over 200 replications for heteroscedastic error model. Bold face indicates the best performance

BART-CQR	QBART (25%)	QBART (50%)	QBART (75%)	BART	CQ regression
**16.623(3.676)**	21.347(3.629)	24.568(4.399)	23.345(5.398)	17.419(4.282)	31.297(4.623)

## Real Data Examples

For the real data analysis, we consider three benchmark/public datasets:

**Ozone Data**: This dataset records ozone levels (in parts per billion) in New York from May to September 1973. The predictors include solar radiation level, wind speed, maximum daily temperature, month, and day of measurement. After removing observations with missing values, we have $n=153$ observations.

**Table 3:** Real data: Test data average RMSE based on 5 folds cross-validation

	BART-CQR	QBART (25%)	QBART (50%)	QBART (75%)	CQ regression
Ozone Data	**16.920**	20.587	23.769	21.327	26.149
Auto Insurance Data	**8.457**	9.352	8.682	9.819	8.584
Boston Housing Data	**0.156**	0.192	0.190	0.189	0.186

**Auto Insurance Data**: This dataset consists of $$ n=2812$$ auto insurance policyholders with 56 predictors along with an aggregate paid claim amount. Examples of predictors include the driver’s age, driver’s income, vehicle use (commercial or non-commercial), vehicle type (one of six categories), and the driver’s gender. The response variable is the aggregate claim amount, which is skewed with a significant number of policyholders having zero claims. For non-zero claims, the reported amounts tend to be larger.

**Fig. 1:**
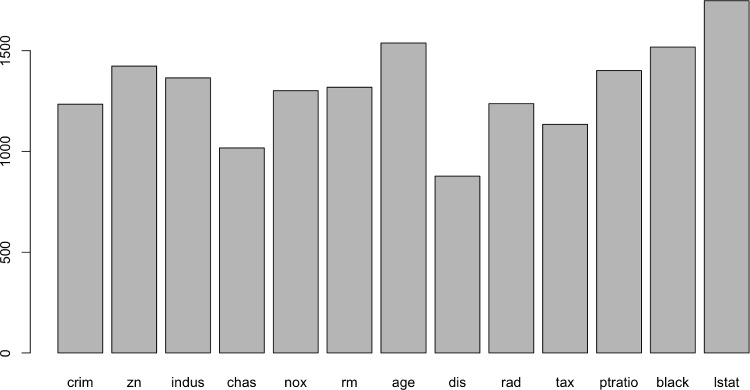
Variable used count in the BART-CQR for Boston housing data

**Boston Housing Data**: This dataset includes $$n=506$$ samples. We examine the relationship between the log-transformed corrected median value of owner-occupied housing (in $1000), denoted as mdev, and 13 explanatory variables: crim (per capita crime rate by town), zn (proportion of residential land zoned for lots over 25,000 sq.ft), indus (proportion of non-retail business acres per town), chas (Charles River dummy variable), nox (nitrogen oxides concentration), rm (average number of rooms per dwelling), age (proportion of owner-occupied units built prior to 1940), dis (weighted mean of distances to five Boston employment centers), rad (index of accessibility to radial highways), tax (full-value property-tax rate per $10,000), ptratio (pupil-teacher ratio by town), lstat (percentage of lower status of the population).

These three datasets are available in the R packages *datasets* (R Core Team 2024), *HDtweedie* (Qian et al. 2022), and *MASS* (Venables and Ripley 2002), respectively. To evaluate predictive performance, we apply 5-fold cross-validation to each dataset. Specifically, the data are randomly partitioned into five nearly equal-sized folds. In each iteration, four of the five folds are used for training, and the remaining fold is used for testing. Predictive RMSE is computed on each test fold, and the average RMSE across all five folds is reported as the final performance measure. As in the simulation study, we set the number of trees to $n_T = 200$, and for the composite quantile methods, we use $K = 9$ quantile levels.

Table 3 summarizes the results. For all data, the BART-CQR provides the smallest RMSE, implying that the proposed method works well with complex real data sets. While the composite quantile regression model may work well if the variables are linearly related, it may collapse under complicated structures. On the other hand, BART models perform well with various structured data, but we need to determine the proper quantile level for the QBART. The proposed composite quantile BART strikes a balance and provides the best performance in these datasets.

For the interpretation, we consider the effect of predictors on the outcome. However, tree models do not directly provide a summary of the effect of a single predictor, or a subset of predictors, on the outcome. We first examine how many times each variable appeared in the collection of trees, which provides a summary similar to the variable importance plot used in boosting and random forests. For simplicity and conciseness of the paper, we report results only for the Boston housing data, among the three real datasets we considered. Figure 1 shows the barplot of the counts in BART-CQR for Boston housing data. We observe that lstat appears most frequently in the trees, highlighting the significant impact of socio-economic status on housing prices.

Furthermore, as suggested by Chipman et al. (2014), we use Friedman’s partial dependence function (Friedman 2001) to summarize the marginal effect due to a subset of the predictors, $\textbf{x} _S$, by aggregating over the predictors in the complement set, $\textbf{x} _C$, i.e., $\textbf{x} = [ \textbf{x} _S, \textbf{x} _C]$. The marginal dependence function is defined by fixing $\textbf{x} _S$ while aggregating over the observed settings of the complement predictors in the data set: $f( \textbf{x} _S) = \frac{1}{n} \sum _{i=1}^n f( \textbf{x} _S, \textbf{x} _{iC} )$.

**Fig. 2:**
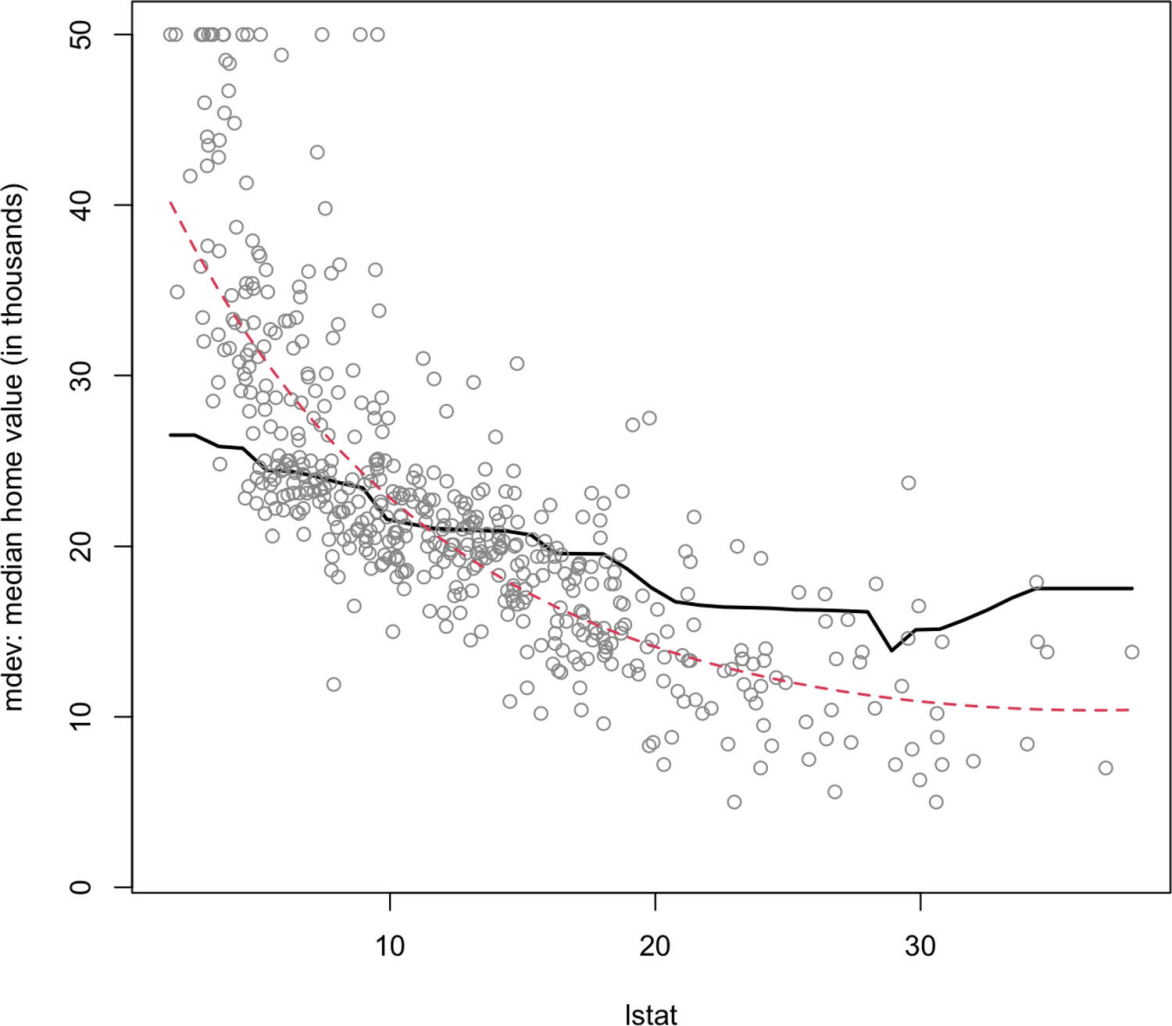
The Boston housing data: the marginal effect of lstat on mdev while aggregating over the other covariates with Friedman’s partial dependence function. The marginal estimate from BART-CQR is shown by the black solid line, and the red dashed line comes from the linear regression model where a quadratic effect of lstat with respect to the logarithm of mdev is assumed

Figure 2 summarizes the marginal effect of lstat on mdev while aggregating over the other predictors with Friedman’s partial dependence function. We observe a negative effect on mdev, shown by the black solid line, which implies that less affluent neighborhoods have lower home values. Compared to the quadratic regression results, shown by the red dashed line, BART-CQR provides a more robust fitted line and also well-captures the complex non-linear relationship between the predictors and the outcome.

## Conclusion and Discussion

In this paper, we proposed a novel Bayesian framework, BART-CQR, which integrates BART with the CQR approach. This method is designed to handle complex, nonlinear relationships and heavy-tailed error distributions, extending the flexibility and robustness of existing quantile-based models. We developed a fully Bayesian hierarchical formulation for BART-CQR and derived an efficient Gibbs sampler for posterior inference. Through comprehensive simulation studies and real data applications, we demonstrated that BART-CQR consistently outperforms classical BART, quantile BART and standard linear composite quantile regression in terms of predictive accuracy. Compared to quantile BART, BART-CQR eliminates the challenge of quantile level selection. Additionally, it offers greater modeling flexibility than standard CQR by capturing nonlinear effects and interactions via tree ensembles. Thus, the proposed BART-CQR method inherits the strengths of both the BART framework and the composite quantile approach, offering a powerful alternative for analyzing complex, non-Gaussian data in moderately sized regression problems with up to approximately 3000 observations.

The proposed approach has a couple of limitations. Although there is room to improve computational efficiency through careful optimization, BART-CQR requires more time than classical BART and quantile BART due to the complexity of the tree ensemble model and MCMC sampling, which may limit its scalability to very large datasets. Moreover, the current method assumes independent and identically distributed errors; its performance under dependent or longitudinal data settings remains to be investigated. In addition, this work does not explore model selection or variable selection properties of BART-CQR, which could be important in high-dimensional settings. Investigating these aspects both theoretically and empirically constitutes an important direction for future research.

Several promising directions exist for future research. Extensions could include incorporating recent advances in BART, such as softBART (Linero and Yang 2018) and BART models with random effects for hierarchical data (Tan and Roy 2019). Furthermore, model selection criteria, theoretical properties of the BART-CQR estimator, and applications to specific domains such as finance, epidemiology, and environmental science warrant further investigation.

## Figures and Tables

**Fig. 3:**
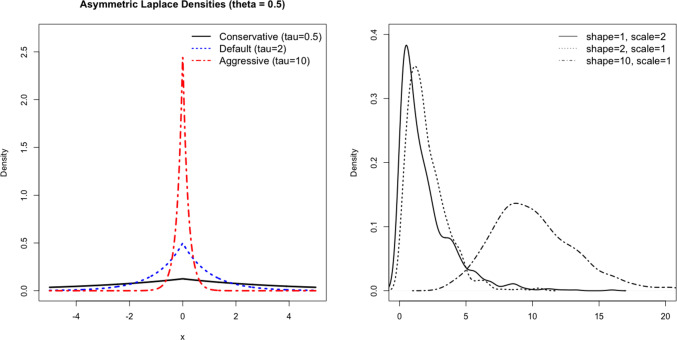
(Left) Density functions of the ALD with $\theta =0.5$ under three different values of the precision parameter $\tau$. (Right) Prior densities of the precision parameter $\tau$ under three different Gamma priors: conservative ($a_\tau =1, b_\tau =2)$, default $(a_\tau =2, b_\tau =1)$, and aggressive ($a_\tau =10, b_\tau =1)$. These priors reflect increasing levels of certainty about the error precision.

## Data Availability

No datasets were generated or analysed during the current study.

## Appendix A Posterior updating scheme

The complete likelihood is

$$\begin{aligned}&f( \textbf{y} | \textbf{X} , \textbf{T} , \textbf{M} , \tau , {\tilde{{\varvec{\nu }}}}, \textbf{b} , \textbf{C} ,{\varvec{\omega }}) \\ &\quad = \prod _{i=1}^n \prod _{k=1}^K \Biggr ( \frac{1}{\sqrt{2\pi \tau ^{-1} \xi _{2k}^2 {\tilde{\nu }}_i} }\Biggr )^{C_{ik}}\\&\quad \exp \Biggr [ - \frac{1}{2} \sum _{i=1}^n \sum _{k=1}^K \frac{ C_{ik} \bigl ( y_i - b_{\theta _k} -\sum _j g( x_i; T_j , M_j) - \xi _{1k} {\tilde{\nu }}_i \bigl )^2 }{ \tau ^{-1} \xi _{2k}^2 {\tilde{\nu }}_i} \Biggr ], \end{aligned}$$

and the posterior updating scheme cycles can be done through the following six posterior draws:

A1 $$\begin{aligned}&f( {\tilde{\nu }}_i | \textbf{T} , \textbf{M} , \textbf{Y} , \textbf{X} , \tau , {\tilde{\nu }}_{(-i)}, \textbf{b} , \textbf{C} ,{\varvec{\omega }}) \end{aligned}$$

A2 $$\begin{aligned}&f( (T_j, M_j ) | \textbf{T} _{(-j)}, \textbf{M} _{(-j)}, \textbf{Y} , \textbf{X} , \tau , {\tilde{{\varvec{\nu }}}}, \textbf{b} , \textbf{C} ,{\varvec{\omega }}) \end{aligned}$$

A3 $$\begin{aligned}&f ( \tau | \textbf{T} , \textbf{M} , \textbf{Y} , \textbf{X} , {\tilde{{\varvec{\nu }}}}, \textbf{b} , \textbf{C} ,{\varvec{\omega }}) \end{aligned}$$

A4 $$\begin{aligned}&f( \textbf{C} _i | \textbf{T} , \textbf{M} , \textbf{Y} , \textbf{X} , \tau , {\tilde{{\varvec{\nu }}}}, \textbf{b} , \textbf{C} _{-i} ,{\varvec{\omega }}) \end{aligned}$$

A5 $$\begin{aligned}&f( {\varvec{\omega }}| \textbf{T} , \textbf{M} , \textbf{Y} , \textbf{X} , \tau , {\tilde{{\varvec{\nu }}}}, \textbf{b} , \textbf{C} ) \end{aligned}$$

A6 $$\begin{aligned}&f( b_{\theta _k } | \textbf{T} , \textbf{M} , \textbf{Y} , \textbf{X} , \tau ,{\tilde{{\varvec{\nu }}}}, b_{-\theta _k } , \textbf{C} , {\varvec{\omega }}). \end{aligned}$$

For (A1), we have $f( {\tilde{\nu }}_i | \textbf{T} , \textbf{M} , \textbf{Y} , \textbf{X} , \tau , {\tilde{\nu }}_{(-i)}, \textbf{b} , \textbf{C} ,{\varvec{\omega }}) \propto \nu _i^{-1/2} \exp \Biggr \{ -\frac{1}{2}\biggl ( \delta _{1i} {\tilde{\nu }}_i^{-1} + \delta _{2i} {\tilde{\nu }}_i \biggl ) \Biggr \} ,$ where $\delta _{1i} := \sum _k \frac{ C_{ik} \left( y_i -b_{\theta _k} - \sum _j g( x_i; T_j , M_j) \right) ^2 }{\tau ^{-1} \xi _{2k}^2}$ and $\delta _{2i}= \sum _k \frac{C_{ik} \xi _{1k}^2}{\xi _{2k}^2 \tau ^{-1}} + 2 \tau$. Therefore, we sequentially samples ${\tilde{\nu }}_i$ from a Generalized Inverse Gaussian distribution.

For (A3), we derive that

$$\begin{aligned} \begin{aligned} f ( \tau&| \textbf{T} , \textbf{M} , \textbf{Y} , \textbf{X} , {\tilde{{\varvec{\nu }}}}, \textbf{b} , \textbf{C} ,{\varvec{\omega }}) \propto \tau ^{n/2+n \!+\! \frac{\alpha }{2}-1} \exp \Bigg \{ \!-\!\tau \biggl [ \!\sum _{i=1}^n\sum _{k=1}^K \frac{ C_{ik}( y_i \!-\! b_{\theta _k} \!-\! \sum _j g( x_i; T_j , M_j)\! -\! \xi _{1k} {\tilde{\nu }}_i )^2 }{ 2 \xi _{2k}^2 {\tilde{\nu }}_i} \!+\! \sum _{i=1}^n {\tilde{\nu }}_i \!+\! b_\tau \biggl ] \Bigg \}, \end{aligned} \end{aligned}$$

which is a Gamma distribution, and for $f( \textbf{C} _i | \textbf{T} , \textbf{M} , \textbf{Y} , \textbf{X} , \tau , {\tilde{{\varvec{\nu }}}}, \textbf{b} , \textbf{C} _{-i} ,{\varvec{\omega }})$ in (A4),

$$\begin{aligned} \begin{aligned}&f( \textbf{C} _i | \textbf{T} , \textbf{M} , \textbf{Y} , \textbf{X} , \tau , {\tilde{{\varvec{\nu }}}}, \textbf{b} , \textbf{C} _{-i} ,{\varvec{\omega }}) \\&\quad \propto \prod _{k=1}^K \Bigg \{ \biggl ( \frac{1}{\sqrt{2\pi \tau ^{-1} \xi _{2k}^2 {\tilde{\nu }}_i} }\biggl )^{C_{ik}}\\&\quad \exp \bigg [ - \frac{1}{2} \frac{ C_{ik} ( y_i - b_{\theta _k} - \sum _j g( x_i; T_j , M_j)- \xi _{1k} {\tilde{\nu }}_i )^2 }{ \tau ^{-1} \xi _{2k}^2 {\tilde{\nu }}_i} \bigg ] \Bigg \} \\&\quad \prod _{k=1}^K \omega _k^{C_{ik}}\propto \prod _{k=1}^K \Bigg \{ \biggl ( \frac{ \omega _k}{ \xi _{2k} }\biggl )\\&\quad \exp \biggl [ - \frac{1}{2} \frac{ ( y_i - b_{\theta _k} - \sum _j g( x_i; T_j , M_j) - \xi _{1k} {\tilde{\nu }}_i )^2 }{ \tau ^{-1} \xi _{2k}^2 {\tilde{\nu }}_i} \biggl ] \Bigg \}^{C_{ik}} , \end{aligned} \end{aligned}$$

which is a Multinomial$(1, {\hat{p}}_1, \ldots , {\hat{p}}_K)$, where

$$\begin{aligned} {\hat{p}}_k = \frac{\Big ( \frac{ \omega _k}{ \xi _{2k} }\Big ) \exp { \bigg [ - \frac{1}{2} \frac{ ( y_i - b_{\theta _k} - \sum _j g( x_i; T_j , M_j) - \xi _{1k} {\tilde{\nu }}_i )^2 }{ \tau ^{-1} \xi _{2k}^2 {\tilde{\nu }}_i} \bigg ] } }{ \sum _k \Bigg \{ \Big ( \frac{ \omega _k}{ \xi _{2k} }\Big ) \exp { \bigg [ - \frac{1}{2} \frac{ ( y_i - b_{\theta _k} - \sum _j g( x_i; T_j , M_j) - \xi _{1k} {\tilde{\nu }}_i )^2 }{ \tau ^{-1} \xi _{2k}^2 {\tilde{\nu }}_i} \bigg ] } \Bigg \} }. \end{aligned}$$

The draw in (A5) is Dirichlet$( n_1 + \alpha _1, \ldots , n_K + \alpha _K)$

$$\begin{aligned} \begin{aligned} f( {\varvec{\omega }}| \textbf{T} , \textbf{M} , \textbf{Y} , \textbf{X} , \tau , {\tilde{{\varvec{\nu }}}}, \textbf{b} , \textbf{C} )&\propto \prod _{k=1}^K \omega _k^{ \alpha _k + n_k} , ~~\text {where} n_k := \sum _i C_{ik}. \end{aligned} \end{aligned}$$

For the intercept (A6),

$$\begin{aligned} \begin{aligned} f( b_{\theta _k }&| \textbf{T} , \textbf{M} , \textbf{Y} , \textbf{X} , \tau ,{\tilde{{\varvec{\nu }}}}, b_{-\theta _k } , \textbf{C} ,{\varvec{\omega }}) \propto \\&\quad \exp \Biggl ( - \frac{1}{2} \sum _i\frac{ C_{ik}}{ \tau ^{-1} \xi _{2k}^2 {\tilde{\nu }}_i} b_{\theta _k }^2 + \sum _i \frac{ C_{ik}{\tilde{r}}_{ik} }{ \tau ^{-1} \xi _{2k}^2 {\tilde{\nu }}_i} b_{\theta _k } \Biggl ) , \end{aligned} \end{aligned}$$

where ${\tilde{r}}_{ik} : = y_i - \sum _j g( x_i; T_j , M_j) - \xi _{1k} {\tilde{\nu }}_i$, and it is a normal distribution.

Now, for the draw (A2), first define

A7 $$\begin{aligned} &  r_{ij} := y_i -b_{\theta _{k}} - \sum _{l \ne j } g( \textbf{x} _i ; T_l , M_l ) - \xi _{1k} {\tilde{\nu }}_i = g( \textbf{x} _i ; T_j , M_j ) \nonumber \\ &  \quad + \tau ^{-1/2}\xi _{2k} \sqrt{ {\tilde{\nu }}_i} z_i , \nonumber \\ &  \quad ~~\text {for}~ y_i \in \text {cluster} ~k. \end{aligned}$$

Then, draw from (A2) is equivalent to draw from a single regression tree $r_{ij}= g( \textbf{x} _i ; T_j , M_j ) + \tau ^{-1/2}\xi _{2k} \sqrt{ {\tilde{\nu }}_i} z_i$ for $i=1, \ldots , n$. In the tree $T_j$, assume that there are $m_j$ terminal nodes and $n_{l}$ observations fall into terminal node *l* for $l=1, \ldots , m_j$ ($n_1 + \ldots + n_{m_j}=n$). Lets consider the set of observations fall into terminal node *l*. Since for each $y_i$, there is corresponding $C_{ik}$ such that $C_{ik}=1$, we can consider a pair $\{(i, k) |~k \text { such that } C_{ik}=1 \}$ for each *i*. Denote the set of (*i*, *k*)’s that fall into terminal node *l* as $G_l$. Further, define $\textbf{r} _j = ( \textbf{r} _{j,1}, \ldots , \textbf{r} _{j,m_j})$ with $\textbf{r} _{j,l}= (r_{1, j, l } , \ldots , r_{n_l ,j,l})$, where $r_{i,j,l}$ be a observation that falls into *l*th terminal node in tree $T_j$ for $i \in G_l$, and we have $r_{i, j,l} \sim N ( \mu _{jl} , \tau ^{-1} \xi _{2k}^2 {\tilde{\nu }}_{i} )$ for $(i,k) \in G_l$. Then the likelihood of the single tree in (A7) is

$$\begin{aligned} &  f( \textbf{r} _j | \textbf{X} , \textbf{T} _{j}, \textbf{M} _{j },\tau ,{\tilde{{\varvec{\nu }}}}, \textbf{b} , \textbf{C} , {\varvec{\omega }})\\ &  \quad = \prod _{l=1}^{m_j} f( \textbf{r} _{j,l} | \textbf{X} _l , \textbf{T} _{j}, \textbf{M} _{j }, \tau ,{\tilde{{\varvec{\nu }}}}_l, \textbf{b} , \textbf{C} , {\varvec{\omega }}), \end{aligned}$$

where

$$\begin{aligned} &  f( \textbf{r} _{j,l} | \textbf{X} _l , \textbf{T} _{j}, \textbf{M} _{j }, \tau ,{\tilde{{\varvec{\nu }}}}_l, \textbf{b} , \textbf{C} , {\varvec{\omega }}) \\ &  \quad = f( \textbf{r} _{j, l} | \mu _{jl}, \tau ,{\tilde{{\varvec{\nu }}}}_l, \textbf{b} , \textbf{C} , {\varvec{\omega }}) \\ &  \quad = \prod _{(i,k) \in G_l} \frac{1}{ \sqrt{2 \pi \tau ^{-1} \xi _{2k}^2 {\tilde{\nu }}_{i} }} \exp \Bigg [ - \frac{1}{2} \frac{ (r_{i, j,l}- \mu _{jl} )^2 }{ \tau ^{-1} \xi _{2k}^2 {\tilde{\nu }}_{i}} \Bigg ]. \end{aligned}$$

Now, the draw from $f( (T_j, M_j ) | \textbf{T} _{(-j)}, \textbf{M} _{(-j)}, \textbf{Y} , \textbf{X} , \tau , {\tilde{{\varvec{\nu }}}}, \textbf{b} , \textbf{C} , {\varvec{\omega }})$ can be done by two successive steps as

A8 $$\begin{aligned} f( T_j&|&\textbf{r} _{j}, \tau , {\tilde{{\varvec{\nu }}}}, \textbf{b} , \textbf{C} , {\varvec{\omega }}) , \end{aligned}$$

A9 $$\begin{aligned} f( M_j&|&T_j, \textbf{r} _{j}, \tau , {\tilde{{\varvec{\nu }}}} , \textbf{b} , \textbf{C} , {\varvec{\omega }}). \end{aligned}$$

For (A8), we use Metropolis-Hastings algorithm. We first derive a formula of $f( T_j | \textbf{r} _{j}, \tau , {\tilde{{\varvec{\nu }}}}, \textbf{b} , \textbf{C} , {\varvec{\omega }})$:

A10 $$\begin{aligned}&f(T_j | \textbf{r} _{j}, \tau , {\tilde{{\varvec{\nu }}}}, \textbf{b} , \textbf{C} , {\varvec{\omega }}) \propto ~ p (T_j)\nonumber \\&\quad \int p( \textbf{r} _{j} | M_j , T_j, \tau , {\tilde{{\varvec{\nu }}}}, \textbf{b} , \textbf{C} , {\varvec{\omega }}) p(M_j | T_j , \tau , {\tilde{{\varvec{\nu }}}}, \textbf{b} , \textbf{C} , {\varvec{\omega }}) dM_j \nonumber \\&\quad = p (T_j) \prod _l \Bigg [ \int \left( \prod _{(i,k) \in G_l} p(r_{i, j,l} | M_j , T_j, \tau , {\tilde{{\varvec{\nu }}}}, \textbf{b} , \textbf{C} , {\varvec{\omega }}) \right) \nonumber \\&\qquad \times p( \mu _{jl} | T_j , \tau , {\tilde{{\varvec{\nu }}}}, \textbf{b} , \textbf{C} , {\varvec{\omega }}) d \mu _{jl} \Bigg ] \nonumber \\&\quad = p (T_j) \prod _l \Biggr [ \int \Biggl \{ \prod _{(i,k) \in G_l} \frac{1}{ \sqrt{2 \pi \tau ^{-1} \xi _{2k}^2 {\tilde{\nu }}_{i} }} \nonumber \\&\quad \exp \bigg [ - \frac{1}{2} \frac{ (r_{i, j,l}- \mu _{jl} )^2 }{ \tau ^{-1} \xi _{2k}^2 {\tilde{\nu }}_{i}} \bigg ] \Biggl \} \frac{1}{ \sqrt{2 \pi \sigma _\mu ^2} }\nonumber \\&\quad \exp \biggl ( -\frac{ \mu _{jl}^2 }{ 2\sigma _\mu ^2} \biggl ) d \mu _{jl}\Biggr ]\nonumber \\&\quad = p (T_j) \prod _l \Bigg \{ \biggl ( \frac{1}{ \sqrt{ 2 \pi \tau ^{-1} }} \biggl )^{n_{l}} \biggl ( \prod _{(i,k) \in G_l} \xi _{2k}^{-1}{\tilde{\nu }}_{i}^{-1/2} \biggl )\nonumber \\&\quad \exp \biggl ( - \frac{1}{2} \sum _{(i,k) \in G_l} \frac{ r_{i, j,l}^2 }{ \tau ^{-1} \xi _{2k}^2 {\tilde{\nu }}_{i}} \biggl ) \nonumber \\&\quad \times \sqrt{ \frac{ \tau ^{-1} }{ \sigma _\mu ^2 \sum _{(i,k) \in G_l} {\tilde{\nu }}_{i}^{-1} \xi _{2k}^{-2} + \tau ^{-1} }}\nonumber \\&\quad \exp \Bigg [ \frac{\sigma _\mu ^2 \biggl ( \sum _{(i,k) \in G_l} r_{i, j,l} {\tilde{\nu }}_{i}^{-1} \xi _{2k}^{-2} \biggl )^2 }{ 2\tau ^{-1} \biggl ( \tau ^{-1} + \sigma _\mu ^2 \sum _{(i,k) \in G_l} {\tilde{\nu }}_{i}^{-1} \xi _{2k}^{-2}\biggl ) } \Bigg ] \Bigg \} . \end{aligned}$$

Then, to draw from $f( T_j | \textbf{r} _{j}, \tau , {\tilde{{\varvec{\nu }}}} , \textbf{b} , \textbf{C} , {\varvec{\omega }})$, we first obtain a new candidate tree $T_j^*$ with a acceptance rate,

$$\begin{aligned} {\tilde{\alpha }}&= \min \left( 1, \frac{f(T_j^* | \textbf{r} _{j}, \tau , {\tilde{{\varvec{\nu }}}}, \textbf{b} , \textbf{C} , {\varvec{\omega }}) q(T_j | T_j^*)}{f(T_j | \textbf{r} _{j}, \tau , {\tilde{{\varvec{\nu }}}}, \textbf{b} , \textbf{C} , {\varvec{\omega }}) q(T_j^* | T_j)}\right) \\&= \min \left( 1, \frac{ p (T_j^*) \int p( \textbf{r} _{j} | M_j^* , T_j^*, \tau , {\tilde{{\varvec{\nu }}}} , \textbf{b} , \textbf{C} , {\varvec{\omega }}) p(M_j^* | T_j^* , \tau , {\tilde{{\varvec{\nu }}}}, \textbf{b} , \textbf{C} , {\varvec{\omega }}) dM_j ~ q(T_j | T_j^*)}{ p (T_j) \int p( \textbf{r} _{j} | M_j , T_j, \tau , {\tilde{{\varvec{\nu }}}}, \textbf{b} , \textbf{C} , {\varvec{\omega }}) p(M_j | T_j , \tau , {\tilde{{\varvec{\nu }}}}, \textbf{b} , \textbf{C} , {\varvec{\omega }}) dM_j ~ q(T_j^* | T_j)}\right) \\&= \min \left( 1, \frac{p ( \textbf{r} _{j} | T_j^*, \tau , {\tilde{{\varvec{\nu }}}}, \textbf{b} , \textbf{C} , {\varvec{\omega }}) p (T_j^*) q(T_j | T_j^*)}{p ( \textbf{r} _{j} | T_j, \tau , {\tilde{{\varvec{\nu }}}} , \textbf{b} , \textbf{C} , {\varvec{\omega }}) p(T_j) q(T_j^* | T_j)}\right) . \end{aligned}$$

The transition kernel $q(\cdot )$ assigns probabilities of 0.25, 0.25, 0.40, and 0.10 to the moves GROW, PRUNE, SWAP, and CHANGE respectively. Compared to quantile BART (Kindo et al. 2016), we use the same transition kernel and priors for the trees, with the only difference being the likelihood function.

For example, in the GROW case, the likelihood ratio can be computed as

$$\begin{aligned}&\frac{p ( \textbf{r} _{j} | T_j^*, \tau , {\tilde{{\varvec{\nu }}}} , \textbf{b} , \textbf{C} , {\varvec{\omega }}) }{p ( \textbf{r} _j | T_j, \tau , {\tilde{{\varvec{\nu }}}}, \textbf{b} , \textbf{C} , {\varvec{\omega }})} = \frac{ \prod _{l \in \text {two children}} \Bigg [ \int \prod _{i \in G_l} p(r_{i, j,l} | M_j , T_j, \tau , {\tilde{{\varvec{\nu }}}}, \textbf{b} , \textbf{C} , {\varvec{\omega }}) p( \mu _{jl} | T_j , \tau , {\tilde{{\varvec{\nu }}}}, \textbf{b} , \textbf{C} , {\varvec{\omega }}) d \mu _{jl} \Bigg ] }{\prod _{l \in \text {parent}} \Bigg [ \int \prod _{i \in G_l } p(r_{i, j,l} | M_j , T_j, \tau , {\tilde{{\varvec{\nu }}}}, \textbf{b} , \textbf{C} , {\varvec{\omega }}) p( \mu _{jl} | T_j , \tau , {\tilde{{\varvec{\nu }}}}, \textbf{b} , \textbf{C} , {\varvec{\omega }}) d \mu _{jl} \Bigg ] } \\&\quad = \frac{(\textrm{A10}) \text { of left child node} \times (\textrm{A10}) \text { of right child node}}{(\textrm{A10}) \text { of parent node}} = \sqrt{ \frac{ \tau ^{-1}( \tau ^{-1}+ \sigma _\mu ^2 B_P ) }{ ( \tau ^{-1} + \sigma _\mu ^2 B_{L} ) ( \tau ^{-1}+ \sigma _\mu ^2 B_R) }}\\&\qquad \times \exp \Bigg \{ \frac{\sigma _\mu ^2 }{2\tau ^{-1} } \left( \frac{ A_L ^2 }{ \tau ^{-1} + \sigma _\mu ^2 B_L } + \frac{ A_R ^2 }{\tau ^{-1} + \sigma _\mu ^2 B_R } - \frac{ A_P ^2 }{ \tau ^{-1} + \sigma _\mu ^2 B_P } \right) \Bigg \} , \end{aligned}$$

where subscript *P*, *L* and *R* denote “parent", “left", and “right" nodes. $B_{P} := \sum _{(i,k) \in G_P} {\tilde{\nu }}_{i}^{-1} \xi _{2k}^{-2}$ and $A_{P} := \sum _{(i,k) \in G_P} r_{i, j,l} {\tilde{\nu }}_{i}^{-1} \xi _{2k}^{-2}$, where $G_{P}$ is the set of (*i*, *k*)s fall into parent node. Similarly define $A_L$, $B_L$, $A_R$, and $B_R$ .

Given an update tree, $\mu _{jl}$, *l*th terminal node parameter at tree $T_j$ in (A9) can be drawn as:

$$\begin{aligned}&f( \mu _{jl} | T_j, \textbf{r} _{j}, \tau , {\tilde{{\varvec{\nu }}}} , \textbf{b} , \textbf{C} , {\varvec{\omega }}) \propto \exp \Bigg [ - \sum _{(i,k) \in G_l} \frac{ (r_{i,j,l} - \mu _{jl})^2 }{ 2 \tau ^{-1} \xi _{2k}^2 {\tilde{\nu }}_{i} } \Bigg ]\\&\quad \exp \Bigg ( - \frac{ \mu _{jl} ^2 }{ 2 \sigma _\mu ^2} \Bigg )\\&\propto \exp \Bigg [ - \bigg (\sum _{(i,k) \in G_l} \frac{ 1 }{{2 \tau ^{-1} \xi _{2k}^2 {\tilde{\nu }}_{i} } } + \frac{1}{ 2 \sigma _\mu ^2} \bigg ) \mu _{jl}^2 \\&\quad + 2\sum _{(i,k) \in G_l} \frac{ r_{i,j,l} }{2 \tau ^{-1} \xi _{2k}^2 {\tilde{\nu }}_{i} } \mu _{jl} \Bigg ], \end{aligned}$$

which is a Gaussian distribution.

## Appendix B Prior Sensitivity Analysis for the Error Precision Parameter $\tau$

As discussed in the main text, BART-CQR assumes an ALD for the error with precision parameter $\tau$ for which we place a Gamma prior:

$$\begin{aligned} \tau \sim ~\tau ^{a_\tau -1} \exp (- b_\tau \tau ). \end{aligned}$$

To assess sensitivity to the hyperparameters $(a_\tau , b_\tau )$, we considered three prior configurations reflecting conservative, default, and aggressive prior beliefs (Figure 3):

- Conservative: ($$a_\tau =1, b_\tau =2)$$
- Default: $$(a_\tau =2, b_\tau =1)$$
- Aggressive: ($$a_\tau =10, b_\tau =1)$$

These choices span a wide range of assumptions regarding the concentration and spread of the ALD errors. Our empirical results suggest that the posterior estimates and predictive performance of BART-CQR remain stable across these prior settings, indicating that the method is not highly sensitive to the calibration of $\tau$ (see Table 4). Therefore, in contrast to standard BART, our model does not require fine-tuned calibration of the prior on $\tau$, although it remains flexible for users who wish to encode informative prior beliefs.

**Table 4:** RMSE of BART-CQR under different Gamma priors on $\tau$, based on simulations using Friedman’s five dimensional test function with various error distributions

	($a_\tau =1, b_\tau =2)$	($a_\tau =2, b_\tau =1)$	($a_\tau =10, b_\tau =1)$
Normal	1.809(0.174)	1.780(0.162)	1.776(0.165)
Contaminated Normal	2.196(0.208)	2.217(0.201)	2.197(0.194)
*t*(2)	1.947(0.196)	1.938(0.209)	1.915(0.213)
ALD ($\theta =0.1)$	8.611(1.004)	8.385(0.987)	8.461(0.949)
ALD ($\theta =0.9)$	8.169(1.065)	7.924(0.910)	7.922(0.94)

## Appendix C Additional Simulation: Effect of Increasing Sample Size under Contaminated Normal Errors

We conducted simulations under the contaminated normal setting using increased training sample sizes. Specifically, we used test sizes of 100, 100, and 200 for training sizes of 200, 400, and 800, respectively. As shown in Table 5, the performance of BART-CQR improves as the sample size increases, with notably lower RMSE at $n=1000$ compared to $n=200$. This trend confirms that the strength of our approach becomes more evident with larger sample sizes. Although performance improves across all methods as the sample size increases, BART-CQR consistently achieves the lowest RMSE across all settings, demonstrating its robustness and efficiency even in larger sample scenarios.

**Table 5:** RMSE (standard error in parentheses) over 100 replications under the contaminated normal setting for increasing training sample sizes (100, 400, and 800). Corresponding test sizes were 100, 100, and 200, respectively

	BART-CQR	QBART (25%)	QBART (50%)	QBART (75%)	BART	CQ regression
$n=200$	**2.712(0.427)**	8.285(1.630)	8.411(1.660)	7.276(1.487)	3.729(0.629)	3.245(0.440)
$n=500$	**1.722(0.185)**	8.580(1.172)	7.680(0.943)	6.309(0.755)	3.333(0.569)	3.050(0.310)
$n=1000$	**1.618(0.245)**	7.549(0.692)	6.151(0.616)	4.868(0.413)	3.206(0.363)	2.864(0.274)
